# Effect of pre and post-transplant body mass index on pediatric kidney transplant outcomes

**DOI:** 10.1186/s12887-022-03344-9

**Published:** 2022-05-21

**Authors:** Safaa M. Abdelrahman, Basma Samir, Eman Abobakr Abd Alazem, Noha Musa

**Affiliations:** 1 Department of Pediatrics, Center of Pediatric Nephrology &Transplantation, Kasr Al Ainy School of Medicine, Cairo University, Cairo, Egypt; 2 Cairo University Children’s Hospital, Cairo University Mounira Pediatric Hospital (Abou El Reeshe), Sayyeda Zeinab, Kasr Al Ainy, PO Box: 11562, Cairo, Egypt; 3 Diabetes, Endocrine and Metabolism Pediatric Unit, Kasr Al Ainy School of Medicine, Cairo University, Cairo, Egypt

**Keywords:** BMI, Obesity, Graft survival, Kidney transplantation

## Abstract

**Introduction:**

Kidney transplantation (KT) has been established as an efficient treatment of end stage renal disease (ESRD) with the advantage of allowing the patient to live a nearly healthy life. We aimed to determine whether pre-transplant body mass index (BMI) affects renal allograft function and survival in pediatric KT recipients.

**Methods:**

cross sectional cohort study included 50 post KT recipients (more than 3 years) with an age range of 10 to 15 years, regularly following at the Kidney Transplantation Outpatient Clinic, Cairo University Children’s Hospital, were subjected to a detailed history and physical examination, laboratory investigation in the form of fasting blood glucose (FBG),oral glucose tolerance test (OGTT), lipid profile, hemoglobin A1c (HbA1c) and microalbuminuria.

**Results:**

Pre- post- kidney transplant BMI has significant positive correlation with graft rejection episodes, HbA1c, FBG, BMI post-KT, total cholesterol, triglycerides, and low-density lipoprotein (*p* < 0.01). There was a statistically significant negative correlation between the mean difference of BMI (post – pre) and graft survival in years (*p* = 0.036). Obese patients displayed lower survival compared with non-obese subjects at 5 years, but this was statistically not significant (*p*-value = 0.165).

**Conclusion:**

obesity is an independent risk factor for graft loss and patient death in kidney transplantation. Careful patient selection with pre-transplantation weight reduction is mandatory to reduce the rate of early post-transplantation complications and to improve long-term outcomes.

## Introduction

Several epidemiologic studies have demonstrated that obesity increases the risk of kidney disease, as well as its progression among diagnosed kidney disease patients [1]. Obesity in children who received kidney transplants has increased over time. Obesity is considered a risk for various health issues in transplanted patients as well as in the general population with elevated BMI including diabetes mellitus and cardiovascular disease (CVD) owing to hyperlipidemia, hypertension, and decreased exercise tolerance [2].

Obese pediatric KT recipients have an increased risk of allograft failure [3, 4]. Several studies have shown adverse graft outcomes in obese children following transplantation [5]. Pre-transplant weight status is an important consideration in transplantation, given what is known about post-transplant weight gain. Studies in adults and children have demonstrated that obesity post-transplant is common, and that pre-transplant elevated BMI is a predictor of weight gain after transplantation [3, 6].

Studies have shown that, in renal transplant, the degree of recipient obesity is directly correlated with increased complications after transplantation and longer operative durations [7]. Obesity can be detrimental to renal allografts as it leads to reduction in glomerular filtration rate, ultimately leading to proteinuria that will eventually lead to obesity-related chronic kidney disease (CKD). Hypertension, impaired glucose tolerance, hyperlipidemia, chronic inflammation within fatty tissues, and atherosclerosis in obese patients helped in progression to chronic allograft failure and reducing graft and patient survival [8, 9].

The aim of our work was to study the impact of BMI (pre and post KT) on post-kidney transplant patient and graft survival in Egyptian children under 18 years old.

## Methodology

The current cross sectional cohort study included fifty kidney transplant children and adolescents (more than 3 years post-KT) aged between 5 and 18 years (both sexes) regularly following at the Kidney Transplantation Outpatient Clinic, Cairo University Children’s Hospital (CUCH). Patients with genetic or endocrinal causes of obesity were excluded from the study. The Study protocol was approved by the Research Ethics Committee of Pediatric Department, Faculty of Medicine at Cairo University. Informed consents were obtained from patient’s legal guardians before enrollment. The study population were subjected to detailed history taking including age, sex, original renal disease, duration of dialysis before KT, duration of KT, rejections episodes and treatment, family history of (obesity [is the presence of obesity in children’s parents, siblings and grandparents] [10], dyslipidemia, coronary heart disease, hypertension or renal diseases), history of exercise and physical activity (Exercise and physical activity is defined as any bodily movement produced by the contraction of skeletal muscles that raises energy expenditure above the resting metabolic rate and is characterized by its modality, frequency, intensity, duration, and context of practice [11],graft failure (defined as the need to initiate renal replacement therapy after transplantation), graft survival. Acute rejection episode is defined as a rise in the serum creatinine of at least 30% from baseline levels and accompanied by clinical symptoms and signs, including fever and oliguria, hypertension & tenderness over the graft [12]. Thorough physical examination was performed including blood pressure, anthropometry (height and weight with calculation of weight SDS, height SDS and BMI) and signs of insulin resistance as acanthosis nigricans.

The body weight was measured using Seca scale. The child’s weight was measured without heavy outer garments and shoes with standing in the center of the platform, and weight was distributed evenly to both feet. The weight was recorded to the nearest 0.1 kg then plotted to the Egyptian Growth charts [13]. The standard deviation score for body weight (SDS-weight) for age and Sex was calculated for each subject using the formula: (individual’s measurement – population mean) / population SD [14]. The height was measured using a Harpenden stadiometer. Children were asked to remove their shoes, heavy outer garments, and hair ornaments then stand with his/her back to the height rule with the back of the head, back, buttocks, calves and heels touching the upright bar of the scale and feet together. The child was asked to look straight with the top of the external auditory meatus (ear canal) at a level with the inferior margin of the bony orbit (cheek bone). The height was measured to the nearest centimeter then plotted to the Egyptian Growth curves [13]. Body mass index was calculated as weight in kilograms divided by the square of height in meters (Wt (kg)/Ht (m)^2^). Obesity was defined as BMI ≥ 95^th^ percentile for age.

Blood pressure (BP) was measured using Dinamap on 3 different occasions over 2 weeks (the mean reading was taken). BP was considered normal if < 90^th^ percentile, borderline hypertension if between 90 and 95^th^ percentile, controlled hypertension if < 95^th^ percentile on treatment and uncontrolled hypertension if BP ≥ 95^th^ percentile with treatment.

### Sample collection

A fasting venous blood sample was collected in the morning for determining of fasting blood glucose level, HbA1c, lipid profile including total cholesterol (TC), low density lipoprotein (LDL), high density lipoproteins (HDL and triglyderides (TGs) in addition to kidney functions (urea and creatinine levels). An oral glucose tolerance test (OGTT) was performed with glucose load equivalent to 75 g of glucose powder solved in water or 1.75 g x weight, not exceeding a total of 75 g. Blood samples were collected before taking the glucose and at 30, 60, 90 and 120 min. Impaired fasting glucose (IFG) was defined as fasting plasma glucose levels between 100 and less than 126 mg\dl. Impaired glucose tolerance (IGT) was defined as two hours post load glucose plasma levels between 140 and 200 mg\dl. HbA1c < 5.7% was normal, values between 5.7% and 6.5% denoted prediabetes, while values ≥ 6.5% denoted diabetes. Dyslipidemia was defined as cholesterol > 200 mg/dl, LDL > 130 mg/dl, TGs > 130 mg/dl, while HDL < 45 mg/dl was considered high risk. Urine analysis was performed to determine micro-albuminuria. Micro-albuminuria was defined as urine albumin ranging from 30 to 300 mg from a morning spot urine sample estimated for 24-h urine collection.

#### Statistical methods

Data were coded and entered using the statistical package SPSS (Statistical Package for the Social Sciences) version 25. Data was summarized using mean, standard deviation, median, minimum and maximum in quantitative data and using frequency (count) and relative frequency (percentage) for categorical data. Comparisons between quantitative variables were done using the non-parametric Mann–Whitney test. For comparing categorical data, Chi square test was performed. Exact test was used instead when the expected frequency is less than 5. *P*-values less than 0.05 were considered as statistically significant.

## Results

The current study included fifty (30 males and 20 females) pediatric living donor KT recipients with a mean age of 12.24 ± 3.04 years and a mean duration of KT of 4.66 ± 1.83 yrs. Sixty percent of them are out of consanguineous marriage with 56% having family history of obesity. History of physical exercise was present only in 18% (*n* = 9) of cases. Etiology of ESRD of our study group was CAKUT in 34% (*n* = 17) of the cases and genetic in 28% of cases. Regarding the biochemical data it was found that Serum TGs were high in 40% of cases (*n* = 20), serum cholesterol was high in 54% of cases (*n* = 27), serum HDL was at high risk in 44% of cases (*n* = 22) and at low risk in 36% of cases (*n* = 18), serum HDL was high in 52% of cases (*n* = 26), HbA1c was prediabetic in 36% of cases (*n* = 18), IFG in 38% of cases (*n* = 19) and IGT in 36% of cases (*n* = 18). Regarding the main treatment for episodes of rejection, 88% (*n* = 44) of the patients were treated with solumedrol and 40% (*n* = 20) with ATG, 22% (*n* = 11) with IVIG and plasma exchange was indicated only in 6% (*n* = 3) of the patients. While we had two patients (4%) received CD 20 Rituximab.

Clinical, and biochemical data of the study group are shown in Tables 1. The mean BMI pre-transplantation was 21.34 ± 4.90 kg/m^2^ and the mean BMI post-transplantation was 28.56 ± 6.27 kg /m^2^.

**Table 1:** Clinical and biochemical data of the study group

		**Mean ± SD/Median (IQR)**	**Range**	
***Clinical data***				
**Age (years)**		12.24 ± 3.04	5–17	
**Episodes of rejection**		3.2 ± 1.4	0–6	
**Weight (kg) Pre KT**		27.1 ± 10.48	12–50	
**Weight Pre KT SDS**		0.46 (-0.68 – 1.77)	-5.34- 4.04	
**Weight (kg) Post KT**		39.46 ± 14.02	22—90	
**Weight Post KT SDS**		0.78 ( 0.35 – 2.15)	-2.6 – 4.1	
**Height (cm) Pre KT**		115 ± 14.5	90- 145	
**Height Pre KT SDS**		-1.34 (-2.84 – -0.33)	-4.59 – 4.97	
**Height (cm) Post KT**		133 ± 17.18	95–157	
**Height Post KT SDS**		-1.49 (-2.31—0.69)	-3.94 -2.49	
**BMI Pre KT (kg/m**^**2**^**)**		21.34 ± 4.9	10–32	
**BMI Pre KT SDS**		1.82 (0.68 – 2.63)	-11.69 – 3.6	
**BMI Post KT (kg/m**^**2**^**)**		28.5 ± 6.27	19- 48	
**BMI Post KT SDS**		2.28 (1.67 – 2.83)	0.22 – 3.91	
**Systolic BP**		121.14 ± 12.8	100–150	
**Diastolic BP**		77.12 ± 9.2	60–95	
*Biochemical data*				
**TGs (mg/dl)**		148.08 ± 72.82	62 – 300	
**Cholesterol (mg/dl)**		219.9 ± 48.89	120 – 330	
**HDL (mg/dl)**		52.78 ± 13.65	27 – 70	
**LDL (mg/dl)**		152.36 ± 30.74	70 – 250	
**FBG (mg/dl)**		96.35 ± 11.2	80–119	
**2 h PPBG (mg/dl)**		130.38 ± 27.9	83–179	
**HbA1c (%)**		5.47 ± 0.45	4.8 – 7	
			**Total (*n* = 50)**	
			**Number**	**percentage**
***Cause of ESRD***				
**Genetic**	FSGS		8	16%
	Cystinosis		1	2%
	Nephronophthisis		5	10%
**CAKUT**	PUV		4	8%
	VUR		9	18%
	Neurogenic bladder		4	8%
**Unknown**	Bilateral atrophic kidney		18	36%
**Malignancy**	Wilm’s tumor		1	2%

^*^BMI Body Mass Index, *SDS* Standard Deviation Score, *KT* Kidney Transplantation, *BP* Blood Pressure, *TG* Triglycerides, *HDL* High Density Lipoprotein, *LDL* Low Density Lipoprotein, *FBG* Fasting Blood Glucose, *PPBG* Post Prandial Blood Glucose, *HbA1c* glycosylated Hemoglobin, *FSGS* Focal Segmental Glomeruloscelreosis, *CAKUT* Congenital Anomalies of the Kidney and Urinary Tract,*PUV* Posterior Urethral Valve,*VUR* Vesicourethral Reflux

Regarding correlation between pre-transplant BMI and different study parameters, a significant positive correlation was detected with episodes of graft rejection, mean SBP, DBP, BMI post-KT, HbA1c, FBG, 2 h PPBG, total cholesterol, TGs and LDL (*p* < 0.01), while a significant negative correlation was detected between BMI and HDL (*p* = 0.009). However, no significant correlation was found between BMI pre-KT and duration of transplantation or graft survival (*p* > 0.05) Table 2.

**Table 2:** Correlation between BMI (pre KT and Post KT) and different study parameters

Variables	BMI pre-transplant		BMI post-transplant	
	**r**	***P*-value**	**r**	***P*-value**
**Age**	0.037	0.79	0.062	0.67
**Duration of dialysis**	-0.0235	0.87	-0.004	0.977
**Duration of transplantation**	0.216	0.13	0.21	0.14
**Number of rejections**	0.364	0.009*	0.513	< 0.001*
**Graft survival**	-0.204	0.16	-0.206	0.149
**BMI pre-KT**	-	-	0.592	< 0.001*
**BMI post-KT**	0.819	< 0.001*	-	-
**Systolic BP**	0.53	< 0.001*	0.63	< 0.001*
**Diastolic BP**	0.51	< 0.001*	0.522	< 0.001*
**cholesterol**	0.41	0.004*	0.356	0.011*
**TG**	0.45	0.001*	0.501	< 0.001*
**LDL**	0.485	< 0.001*	-0.398	0.004*
**HDL**	-0.362	0.009*	0.698	< 0.001*
**HbA1C**	0.538	< 0.001*	0.579	< 0.001*
**FBG**	0.553	< 0.001*	0.602	< 0.001*
**2 h PPBG**	0.555	< 0.001*	0.623	< 0.001*

*BMI* Body Mass Index, *SDS* Standard Deviation Score, *KT* Kidney Transplantation, *BP* Blood Pressure, *TG* Triglycerides, *HDL* High Density Lipoprotein, *LDL* Low Density Lipoprotein, *FBG* Fasting Blood Glucose, *PPBG* Post Prandial Blood Glucose, *HbA1c* Glycosylated Hemoglobin

*p-value less than 0.05 and it is statistically significant

A comparison of baseline characteristics between obese and non-obese recipients according to pre-KT BMI are shown in Table 2. A statistically significant difference was detected between both groups regarding acute rejection (*p* = 0.001), chronic rejection (*p* = 0.05), urinary ACR (*p* = 0.002), FBG (*p* < 0.001), 2 h PPBG (*p* < 0.001), HbA1c (*p* = 0.001), TC (*p* = 0.006), TGs (*p* = 0.013) and LDL (*p* = 0.002).

Regarding correlation between post-KT BMI and different study parameters, a significant positive correlation was detected BMI and episodes of graft rejection, SBP, DBP, BMI pre-KT, HbA1c, FBG, 2 h PPBG, TC, TGs and LDL (*p* < 0.01). A significant negative correlation was detected between post-KT BMI and HDL (*p* = 0.004, *r* =—0.398). However, no significant correlation was found between post-KT BMI and duration of transplantation or graft survival (*p* > 0.05).

Comparing obese to non-obese cases (regarding post-transplantation BMI) showed statistically significant difference in age (*p* = 0.001) acute rejection (*p* = 0.026), chronic rejection (*p* = 0.009), graft survival (*p* = 0.047), hypertension (*p* = 0.019), urinary ACR (*p* < 0.001), FBG (*p* = 0.005), 2 h PPBG (*p* = 0.007), TC (*p* = 0.032), LDL(*p* = 0.001), HDL (*p* < 0.001) and HBA1c (*p* = 0.003) shown in Table 3.

**Fig. 1:**
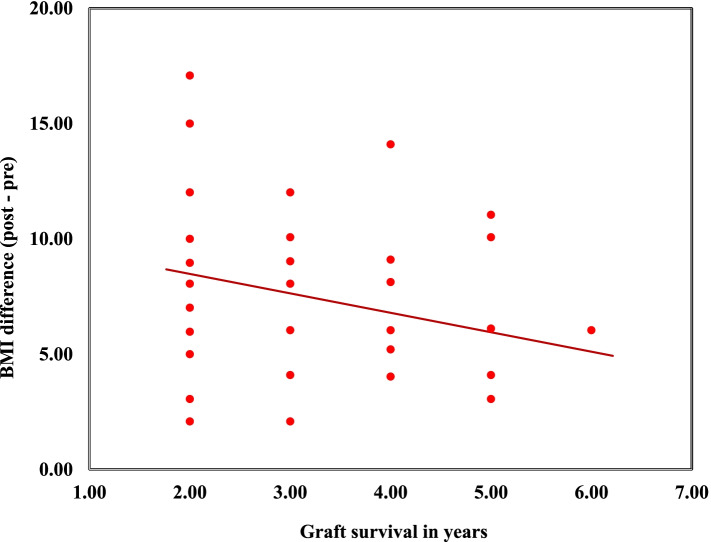
Correlation between mean difference in BMI (post–pre KT) and graft survival. (p: 0.036, r: -0.297)

In the current study, there was a statistically significant increase in the percentage of obesity (BMI > 30) within recipients; 14 cases (28%) were obese pre-transplantation while post transplatation, the prevalence of obesity became 76% (*n* = 38) (*p* < 0.001). A statistically significant negative correlation was detected between the mean difference of BMI (post – pre) and graft survival in years (*p* = 0.036, r-value: -0.297) as well as a statistically significant positive correlation with duration of transplantation (*p* = 0.017, *r* = 0.337) and number of pulse steroids taken (*p* = 0.003,*r* = 0.412) (Fig. 1). One of our cases died 1 month post-transplantation due to fulminant sepsis. Graft loss occurred in two patients due to renal vein thrombosis (Table 3).

**Table 3:** Comparison between Obese and non-obese (pre and post KT) regarding different study parameters

	**BMI post-KT**		***P*-value**	**BMI pre-KT**		***P*-value**
	**Non obese (*n* = 12)**	**Obese (*n* = 38)**		**Non obese (*n* = 36)**	**Obese (*n* = 14)**	
	**No. (%) / Mean ± SD**	**No. (%) / Mean ± SD**		**No. (%) / Mean ± SD**	**No. (%) / Mean ± SD**	
**Sex**						
Male	5 (41.7)	25 (65.8)	0.137	22 (61.1)	8 (57.1)	0.797
Female	7 (58.3)	13 (34.2)		14 (38.9)	6 (42.9)	
**Age (years)**						
5–10	6 (50)	2 (5.3)	0.001٭	7 (19.4)	1 (7.1)	0.102
10–15	6 (50)	27 (71.1)		25 (69.5)	8 (57.1)	
> 15	0 (0)	9 (23.7)		4 (11.1)	5 (35.8)	
**Residence**						
Urban	5 (41.7)	26 (68.4)	0.096	22 (61.1)	9 (64.3)	0.836
Rural	7 (58.3)	12 (31.6)		14 (38.9)	5 (35.7)	
**Acute rejection › 6 ms**						
No	2 (16.7)	3 (7.9)	0.377	4 (11.1)	1 (7.1)	0.675
Yes	10 (83.3)	35 (92.1)		32 (88.9)	13 (92.9)	
**Acute rejection < 6 ms**						
No	12 (100)	26 (68.4)	0.026٭	32 (88.9)	6 (42.9)	0.001٭
Yes	0 (0)	12 (31.6)		4 (11.1)	8 (57.1)	
**Chronic rejection**						
No	12 (100)	23 (60.5)	0.009٭	28 (77.8)	7 (50)	0.05*
Yes	0 (0)	15 (39.5)		8 (22.2)	7 (50)	
**Graft survival**						
< 5 yrs	10 (83.3)	29 (76.3)	0.047*	26 (72.2)	13 (92.9)	0.114
> 5 yrs	2 (16.7)	9 (23.7)		10 (27.8)	1 (7.1)	
**HTN pre- transplantation**						
No	12 (100)	25 (65.8)	0.019٭	29 (80.6)	8 (57.1)	0.090
Yes	0 (0)	13 (34.2)		7 (19.4)	6 (42.9)	
**HTN post-transplantation**						
No	8 (66.7)	18 (47.4)	0.243	21 (58.3)	5 (35.7)	0.151
Yes	4 (33.3)	20 (52.6)		15 (41.7)	9 (64.3)	
**Impaired FBG**						
No	12(100)	19 (50)	0.005٭	30 (83.3)	2 (14.3)	< 0.001٭
Yes	0 (0)	19 (50)		6 (16.7)	12 (85.7)	
**Impaired OGTT**						
No	12(100)	20 (52.6)	0.007٭	29 (80.5)	2 (14.3)	< 0.001٭
Yes	0 (0)	18 (47.4)		7 (19.5)	12 (85.7)	
**Urinary ACR**						
Normal	10 (83.3)	3 (7.9)	< 0.001٭	24 (67.7)	5 (35.7)	0.002*
High	2 (16.7)	35 (92.1)		12 (33.3)	9 (64.3)	
**Cholesterol (mg/dl)**	193.75 ± 31.94	228.16 ± 50.7	0.032٭	208.31 ± 45.61	249.71 ± 45.59	0.006٭
**TG (mg/dl)**	124.08 ± 20.13	155.66 ± 81.58	0.193	132.42 ± 34.68	188.36 ± 119.67	0.013٭
**LDL (mg/dl)**	127.42 ± 31.15	160.24 ± 26.41	0.001٭	144.42 ± 30.66	172.79 ± 20.17	0.002٭
**HDL (mg/dl)**	65.08 ± 15.23	48.89 ± 10.64	0.000٭	55 ± 14.17	47.07 ± 10.64	0.065
**HbA1c (%)**	5.13 ± 0.2	5.58 ± 0.46	0.003٭	5.31 ± 0.3	5.89 ± 0.44	0.001

*BMI* Body Mass Index, *SD* Standard Deviation, *KT* Kidney Transplantation, *FBG* Fasting Blood Glucose, *TG* Triglycerides, *OGTT* Oral Glucose Tolerance test, *ACR* Albumin Creatinine Ratio, *HDL* High Density Lipoprotein, *LDL* Low Density Lipoprotein, *HbA1c* Glycosylated Hemoglobin

*p-value less than 0.05 and it is statistically significant

## Discussion

The epidemic of obesity has a profound effect on many potential transplant recipients. The impact of BMI on post‐transplant outcomes has not been well studied in the pediatric patients undergoing renal transplantation [15].

The present study showed that the mean BMI pre-transplantation was 21.34 ± 4.90 while the mean BMI post-transplantation was 28.56 ± 6.27 with statistically significant difference. This came in agreement with a study done by Mitsnefes et al. who reported in a small study of children that the rate of obesity doubled in the first-year post-transplant [3]. Factors that may contribute to pre- and post-transplant weight gain included reduced exercise capacity due to chronic kidney disease-related fatigue and reduced muscle strength, the metabolic effect of steroids and other medications, and sedentary lifestyle. Also Liu et al., showed that prolonged corticosteroid therapy commonly causes weight gain and redistribution of adipose tissue [16].

### BMI pre-transplantation and dyslipidemia

In the current study population, 40% had hypertriglyceridemia, 54% had hypercholesterolemia, 52% had high LDL and 44% had low HDL. Our results were greatly similar to Sanad and Gharib study who reported that 30% had high triglyceride, 24.7% had high LDL, and 20.7% had low HDL [17]. Dyslipidemia is the most common and consistent abnormality in obese subjects. These lipid abnormalities are typical features of the metabolic syndrome and may be linked to a pro-inflammatory gradient which may originate in the adipose tissue itself and directly affect the endothelium [18].

### BMI pre transplantation and proteinuria

Proteinuria is a powerful predictor of progression of graft dysfunction in KTR [19]. However, the relationship between obesity, proteinuria, and reduced graft survival in KTR is currently unknown. Our results showed a statistically significant direct correlation between BMI pre transplantation and proteinuria (*p* = 0.002). This can be explained with the fact that proteinuria is a marker of renal involvement in obesity that usually precedes GFR decline by several years due to hemodynamic changes that result in glomerular hypertrophy and hyperfiltration [17].

### BMI pre-transplantation and rejection

Acute rejection in the 1^st^ year post-transplantation was more prevalent in our study population. Our results were similar to Meyer‐Kriesche et al. who showed that extremes of body weight were associated with worse graft survival [20]. Hanevold reviewed the NAPRTCS and assessed the impact of pre-transplant obesity on long- term graft survival and demonstrated that there was no significance in overall patient and graft survival. However, they did show that obese children had higher rates of graft loss secondary to thrombosis and also, had higher risk of death secondary to cardiopulmonary disease [5]. The association of obesity with graft failure may be related to immunologic and non-immunologic factors.

### BMI pre-transplantation and graft survival

Our study showed that the five-year graft survival in the non-obese cases was statistically significant than obese cases and, that is came in agreement with a study done by Kaur et al., who found that pediatric transplant recipients with BMIs in the obese range were more likely to experience DGF, acute rejection prolonged hospitalization, and were at a higher risk for graft failure and death, while underweight pre-transplant status was protective for certain age groups [21]. Also, Winnicki et al. showed that obese recipients had an increased risk of graft failure compared to normal weight recipients [4]. On the contrary, Dick et al. found no difference in mortality, graft survival, or DGF among children < 18 years classified as severely thin, normal weight, and severely obese using World Health Organization (WHO) criteria which came in contrast to our results [22]. Mitsnefes et al. as well reported that obese children had an equivalent risk of developing acute rejection as normal weight children in the first year post-transplant [3].

### BMI pre-transplantation and impaired glucose tolerance

We observed in our study that the risks of obesity were progressive with increasing BMI and that there was significant difference between obese and non-obese groups regarding FBG and OGTT. The mechanisms are unclear, but adiposity is known to secrete growth and pro-survival factors. But once the adipose gets to obese state, it shifts toward a pro-inflammatory profile that can worsen graft survival [23].

### BMI and post transplantation rejection and graft survival

In our study, there was statistically significant difference between post-transplant obese and non-obese recipients regarding graft survival, number of graft rejections and chronic rejections. There was a statistically significant negative correlation between the mean difference of BMI (post – pre) and graft survival in years (*p* = 0.036), also there was statistically significant positive correlation between BMI difference and duration of transplantation (*p* = 0.017) and number of pulse steroids taken (*p* = 0.003). However, Beladi Mousavi et al. reported that obesity has significantly increased after KT (*P* = 0.043) being more frequent in those with < 3-year graft duration (*P* = 0.013) and hypertriglyceridemia (*P* = 0.005) without being associated with pretransplant hyperlipidemia, CCD/GFR, proteinuria, and hypertension [24]. On the contrary, Tremblay et al., concluded that BMI was not associated with increased risk of graft loss or recipient death, including when comparing deceased and living donors. Because of the associated co morbidities and increased risk of adverse outcomes following transplantation, some centers have excluded patients with a high BMI (e.g., ≥ 30 kg/m2) from transplantation [25]. Nevertheless, a report by Gill et al. showed that there was a survival benefit for obese patients receiving kidney transplantation compared to dialysis [26].

## Conclusion

BMI impacts early and long‐term renal transplant outcome. Obesity increased the risk of prolonged hospitalization and treatment for acute rejection in the post‐operative period, adversely affecting overall graft survival. Mean increase in BMI post-transplantation affected graft survival.

### Limitation of the study

The relatively small number of patients as pediatric kidney transplant recipient’s number is not high.

## Data Availability

All data will be available upon request from the corresponding author after all authors’ approval.

## Abbreviations

KT: Kidney transplantation
ESRD: End stage renal disease
BMI: Body mass index
FBG: Fasting blood glucose
OGTT: Oral glucose tolerance test
HbA1c: Hemoglobin A1c
CVD: Cardiovascular disease
CKD: Chronic kidney disease
SDS: Standard deviation score
BP: Blood pressure
TC: Total cholesterol
LDL: Low density lipoprotein
HDL: High density lipoproteins
TGS: Triglyderides
IFG: Impaired fasting glucose
IGT: Impaired glucose tolerance
ACR: Albumin creatinine ratio
DGF: Delayed graft function
